# Shape predicts osteoarthritis progression in the first carpometacarpal joint

**DOI:** 10.3389/fbioe.2026.1800839

**Published:** 2026-08-19

**Authors:** Wan M. R. Rusli, Ana Bandarrinha Brandão, Joseph J. Crisco, Angela E. Kedgley

**Affiliations:** 1 Department of Bioengineering, Imperial College London, London, United Kingdom; 2 College of Engineering, International University of Science and Technology in Kuwait (IUK), Ardiya, Kuwait; 3 Department of Mechanical Engineering, Instituto Superior Técnico, Lisboa, Portugal; 4 Department of Orthopaedics, The Warren Alpert Medical School of Brown University and Brown University Health, Providence, RI, United States

**Keywords:** bone morphology, disease progression, first carpometacarpal joint, osteoarthritis, statistical shape modelling

## Abstract

**Introduction:**

Statistical shape modelling enables global geometric features of bones to be quantified and previously has demonstrated differences between healthy and osteoarthritic populations, including at the first carpometacarpal (CMC) joint. However, no study has examined how the variability in the shapes of the metacarpal and trapezium change with time. The aim of this study was to determine if baseline morphological differences in the first metacarpal and trapezium distinguish individuals with progressive first CMC joint osteoarthritis (OA) from non-progressors. It was hypothesized that distinct morphological differences exist in these bones between participants with first CMC joint OA who experience disease progression and those who do not.

**Methods:**

Longitudinal three-dimensional (3D) meshes from 85 osteoarthritic first carpometacarpal (CMC) joints (46 females and 39 males; 57.3 ± 8.4 years) were categorized as non-progressors (n = 49) or progressors (n = 36). Statistical shape modelling quantified morphological changes between baseline and follow-up (1.5 or 6 years) scans for the first metacarpal and trapezium. A non-parametric combination test was used to identify significant morphological differences between time-points.

**Results:**

Morphological differences were identified in both the first metacarpal and trapezium between participants with OA of the first CMC joint that progressed and those that did not. On the first metacarpal, the articulation surface tilt angle and protrusion at the volar beak were two morphological parameters that differed between the progressor and non-progressor groups. On the trapezium, depressions at the volar and radial regions of the articulating surface, and protrusions at the radial and ulnar borders of the carpometacarpal facet were indicated by the shape models for the progressor group.

**Discussion:**

These findings may be used to stratify treatment approaches for CMC joint OA, avoiding unnecessary interventions for those who are unlikely to experience disease progression, while monitoring those who are more likely to experience progression more closely.

## 1 Introduction

The first carpometacarpal (CMC) joint, where the first metacarpal and trapezium bones meet, has a biconcave-convex saddle shape that provides little bony stability, but permits the large range of motion of the thumb. The first CMC joint is a common site for hand osteoarthritis (OA). When compared to interphalangeal joint OA, more pain and functional disability are associated with first CMC joint OA (Bijsterbosch et al., 2010).

In an effort to understand the reason for the high prevalence of first CMC joint OA, many morphological analyses of the articulating surfaces of the joint have been carried out in order to better understand its function (Kuczynski, 1974; Ateshian et al., 1992; Kovler et al., 2004; Marzke et al., 2012; Halilaj et al., 2014b). It is important to measure not just regional morphological patterns, but also the global morphological pattern and this can be done using statistical shape modelling (SSM) (Young and Frangi, 2009). SSM has been used to analyse the complete structure of both the first metacarpal and trapezium in three dimensions (van de Giessen et al., 2011; Schneider et al., 2015; Schneider et al., 2018; Rusli and Kedgley, 2020).

Using SSM, the aspect ratio of the first metacarpal, defined as the ratio between the length of the first metacarpal and its epiphysis diameter, has been shown to be the morphological parameter that exhibits the greatest variance between men and women (Schneider et al., 2015). Furthermore, this anatomical feature has been identified as differing between healthy individuals and patients with first CMC joint OA (Schneider et al., 2018). It has been agreed that the width of the trapezium is greater in first CMC joint OA patients than in the healthy population (Schneider et al., 2018; Ladd et al., 2015). A longer first metacarpal volar beak also has been observed in a population with first CMC joint OA, as compared with a healthy population (Schneider et al., 2015). Using localized component analysis, the first metacarpal facet on the trapezium was observed to be flatter in the osteoarthritic population compared to the healthy population (van de Giessen et al., 2011).

Although SSM has been used to determine the morphological variability of the first metacarpal and trapezium in both healthy and osteoarthritic populations, no study has examined how the variability in anatomical traits changes in a population with time. Using SSM, previous studies on the hip determined that several anatomical traits of the femur can increase the risk of onset and progression of hip osteoarthrosis and possibly lead to total hip replacement (Barr et al., 2012; Barr et al., 2018; Ahedi et al., 2017; van Buuren et al., 2021). The same conclusion was reached for the tibiofemoral (Dube et al., 2018; Gregory et al., 2020) and patellofemoral joints (Liao et al., 2021). However, despite the common occurrence of OA at the joint, no such analysis has been performed for the base of the thumb.

The aim of this study was to determine whether there are differences in morphology between individuals with first CMC joint OA that progresses, those with OA that does not progress, and those without radiographic evidence of CMC OA. In order to achieve this aim, two objectives were set; these were 1) to quantify and compare the morphological variability of the first CMC joint in individuals with early-stage OA, distinguishing between those whose disease progressed and those whose disease did not, and 2) to qualitatively describe the morphology of the first metacarpal and trapezium for progressors and non-progressors at different stages of OA. The hypotheses were that there would be differences in joint morphology between all three groups in this study and that there would be differences in joint morphology between the baseline and follow-up timepoints for those with OA, but not for those without.

## 2 Materials and methods

### 2.1 Participants

Anonymised CT scans of a total of 85 participants from an Institutional Review Board (IRB)-approved observational study, which acquired scans of volunteers with early stage first CMC joint OA over a 6-year period (46 females and 39 males; 57.3 ± 8.4 years), were used in this study (Halilaj et al., 2013; Halilaj et al., 2014c; Halilaj et al., 2014a; Halilaj et al., 2015b; Halilaj et al., 2015a; Crisco et al., 2015a; Halilaj et al., 2015b; Halilaj et al., 2019; McQuillan et al., 2016; 2018; Schneider et al., 2018; Ajayi et al., 2025). IRB approvals were obtained at both Rhode Island Hospital in Providence, RI, United States and Stanford University, CA, United States. Participants were classified as having early-stage first carpometacarpal (CMC) joint OA based on clinical evidence of joint-specific pathology, including pain, localized tenderness, a positive grind test, and/or crepitus. Radiographic inclusion required absent or early-stage OA findings (modified Eaton stages 0–II). Exclusion criteria included any history of trauma, inflammatory arthritis, or surgical intervention capable of altering joint morphology.

### 2.2 CT scanning and 3D surface mesh

Dominant hands were scanned at two time points (baseline and 1.5 and/or 6-year follow-up) with the thumb braced in neutral alignment (Rolyan® Original, Patterson Medical, Bolingbrook, IL). A 16 -slice scanner (GE LightSpeed 16, GE Medical, Milwaukee, WI) with settings of 80 kVp and 80 mA was used. A reconstruction diameter of 200 mm provided an in-plane resolution of 0.391 mm and slice thickness of 0.625 mm.

First metacarpals and trapezia were segmented using density-based thresholding and manual editing in MIMICS (v12-20, Materialise, Leuven, Belgium). To preserve morphology, masks were exported as closed triangular meshes without triangle refinement, utilising a single smoothing iteration. All left hands were mirrored for consistent analysis.

### 2.3 Dataset

**FIGURE 1:**
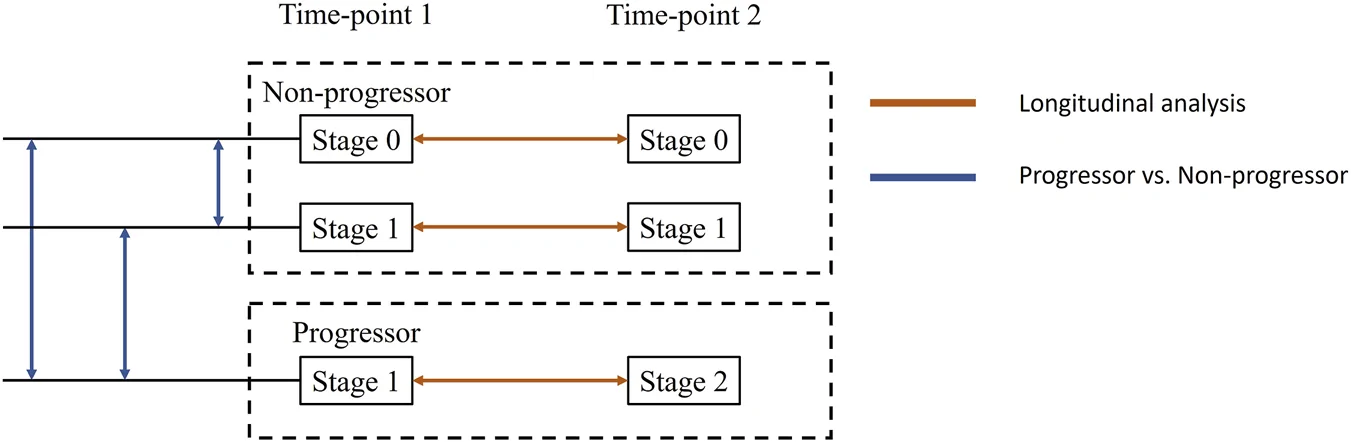
Flowchart depicting the longitudinal study design, group classification, and statistical analysis pathways. Participants were evaluated during an initial baseline scan (time-point 1) and a subsequent follow-up assessment (time-point 2), which occurred at discrete intervals of either 1.5 or 6 years. Clinical staging and osteophyte progression thresholds dictated the group classification. Longitudinal analysis (orange arrows) evaluated intra-group morphological changes across both time-points, whereas the inter-group cross-sectional pathway used baseline data (blue arrows) to determine morphological variations between the non-progressors and progressors.

Participants were split into non-progressor and progressor groups (Figure 1). In both groups, participants were imaged at two-time intervals, which were designated as time-point 1 (baseline scan) and time-point 2 (1.5 or 6 years after the baseline scan). The participants were included in the progressive group if they demonstrated an osteophytes volume exceeding 150 mm^3^ at any time point or a growth rate exceeding 14.6 mm^3^/year. Conversely, participants remaining below these thresholds were included in the non-progressor group (Morton et al., 2022). The numbers of samples in each dataset were 22, 27 and 36 for stage 0 OA (non-progressor), stage 1 OA (non-progressor) and stage 1 OA (progressor), respectively.

### 2.4 Statistical shape modelling

#### 2.4.1 Statistical shape model

**FIGURE 2:**
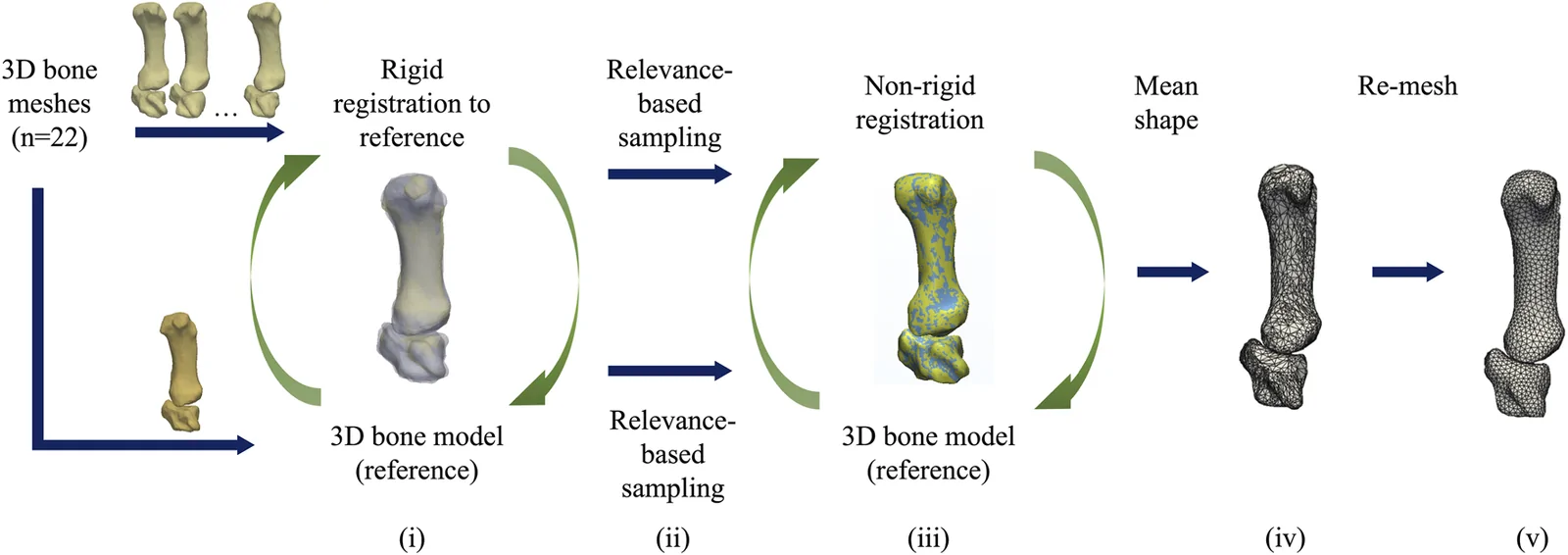
The computational pipeline used in the construction of the initial reference template. The baseline template was derived from twenty-two 3D meshes, which captured the first metacarpal and trapezium of the stage 0 non-progressors at time-point 1. These samples underwent a multi-step spatial registration process. (i) Initial rigid coherent point alignment was followed by (ii) relevance-based sampling. Then, (iii) non-rigid deformation established the point-to-point correspondence, resulting in (iv) the Fréchet mean shape. It was then subjected to uniform isotropic re-meshing with (v) 1,298 and 587 vertices for the first metacarpal and trapezium, respectively.

Scans from the non-progressor group, which were classified as stage 0 OA, represented healthy first metacarpals and trapeziums; thus, an initial template (*T*_*i*_) was constructed from this dataset at time-point 1 (Figure 2). The meshes were aligned to a set of reference 3D meshes, which were selected randomly from the non-progressor stage 0 group, using the rigid coherent point drift algorithm (Myronenko and Song, 2010). A coarse non-rigid registration pipeline was implemented to obtain point-to-point correspondences between all the samples in the group (Rusli and Kedgley, 2020). The mean 3D meshes of the first metacarpal and trapezium were calculated and re-meshed in Geomagic Design X (3D Systems, Research Triangle Park, NC, United States) to have a uniform distribution of points across bones. The *T*_*i*_ with 1,298 and 587 points for the first metacarpal and trapezium respectively, was used as the initial reference 3D meshes in all the datasets to obtain the shape model of both the first metacarpal and trapezium.

In order to create the statistical shape models, all groups of meshes used in this study were aligned to the *T*_*i*_ using the rigid coherent point drift algorithm (Myronenko and Song, 2010). Acting as the initial reference, *T*_*i*_ was then used to determine the reference 3D meshes using the Fréchet mean of each dataset to eliminate any bias caused by the use of *T*_*i*_ (Durrleman et al., 2014). Using a large deformation diffeomorphic metric mapping framework, the deformation of the reference 3D meshes for each of the groups to all samples in their respective sub-groups was estimated based on the convolution between the control points and their momenta and the vertices of the samples 3D meshes in the datasets (Durrleman et al., 2014). Projection pursuit principal component analysis (Croux et al., 2007) was used to obtain the mean and its principal components of the momenta for each of the datasets and these momenta were used to construct the mean shape model and its principal components at ±2 standard deviations of the first metacarpal and trapezium. A leave-one-out analysis was carried out to evaluate the generalisability of the developed statistical shape model. A dimensionality reduction threshold of 5% was applied to the obtained principal components (van de Giessen et al., 2009). This was implemented to isolate meaningful macro-structural variations from high-frequency imaging noise and minor surface artifacts during geometric evaluation. Hence, only principal components explaining over 5% of total morphological variance were retained.

#### 2.4.2 Statistical analysis

A Chi-Square test of independence was implemented to evaluate the longitudinal group distribution. Because the follow-up intervals varied amongst participants, spanning either 1.5 or 6 years, this statistical test was done as a precaution to mitigate the risk of inherent temporal selection bias. The analysis was used to determine whether temporal duration remained fundamentally independent of the assigned groups, which explicitly included non-progressor stage 0, non-progressor stage 1, and progressor stage 1 groups.

A non-parametric combination test was implemented to statistically determine the morphological differences between groups and at different time points at the coordinate level and landmark level (Pesarin and Salmaso, 2010). The non-parametric combination test was chosen because the normality assumption was not met due to the high dimensional dataset used in this study. This method allowed for the evaluation of whether the morphology of the 3D meshes of the first metacarpal and trapezium resulting from the statistical shape modelling methods were different between time-point 1 and time-point 2 for all datasets. Partial test statistics at the coordinate level were used to analyse the x, y and z coordinates individually. By combining these partial tests, the p-value for each of the points that represented the morphology of both the first metacarpal and trapezium were determined. In an attempt to avoid false positives, since multiple hypotheses were tested, both the p-value and family-wise error rate p-value were determined at each level of hypothesis testing (coordinate and landmark levels). Closed testing was done at the landmark level, producing a global family-wise error rate p-value and only this global family-wise error rate p-value was reported.

#### 2.4.3 Shape analysis

Visual inspection was carried out on each group using their respective mean model based on the statistical results from Section B (II). A surface-to-surface distance comparison was conducted between the mean models of the progressor group between time-points 1 and 2. Following this, the same comparison method was used between the mean model of the first CMC joint at time-point 1 across all groups to observe the differences between the geometries representing OA progression and non-progression.

## 3 Results

The Chi-Square contingency analysis (Table 1) indicated that the follow-up intervals of 1.5 and 6 years remained independent of the assigned groups (p = 0.257). This outcome suggested a minimisation of temporal selection bias, meaning that any observed osteoarthritis progression may reflect true pathological morphology rather than a confounding artifact generated by differences in temporal gaps between imaging.

**TABLE 1:** Contingency table of osteoarthritis status against follow-up interval. Based on this contingency table, the Chi-Square test of independence indicates that the follow-up intervals remained independent of the assigned groups (p = 0.257).

Group	1.5-year follow-up	6-year follow-up	Total
Non-progressor Stage 0	12	10	22
Non-progressor Stage 1	19	8	27
Progressor Stage 1	27	9	36
Total	58	27	85

**FIGURE 3:**
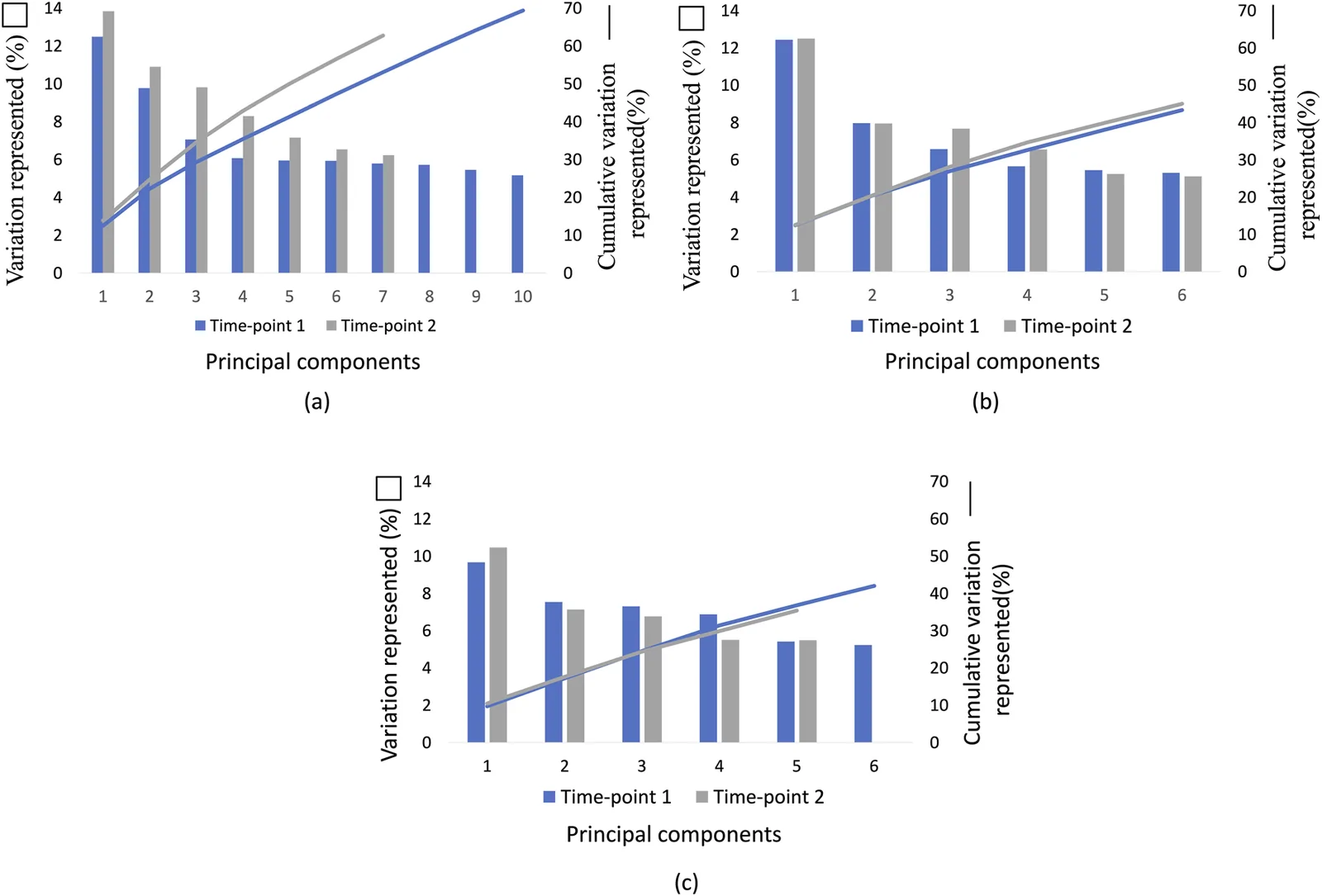
This principal component (PC) analysis details both the separate (bars) and cumulative (lines) morphological variations observed within the first carpometacarpal joint groups across their longitudinal follow-up intervals. Baseline data (blue) were compared against the follow-up time-point 2 (grey). Only PCs explaining over 5% of the total morphological variance are displayed. **(a)** The non-progressor stage 0 group demonstrated a change from 10 dominant PCs at time-point 1 to seven at time-point 2. **(b)** Variation patterns within the non-progressor stage 1 group appear to remain stable over time. **(c)** The progressor stage 1 group demonstrated a shift in morphological variation from six dominant PCs at time-point 1 down to five at time-point 2.

The number of principal components explaining over 5% of morphological variance differed across groups and time points. A leave-one-out analysis is a common method to evaluate whether the developed shape model is able to capture true population variation and not the noise or overfitting (Grant et al., 2020; Nolte et al., 2020). The root mean square error (RMSE) obtained from this leave-one-out analysis was 0.45 mm. Because this RMSE aligns with the voxel dimensions of the source imaging, the model generalises well and consistent with the resolution limits of the source imaging. For the non-progressor group with stage 0 OA (Figure 3a), ten principal components met this threshold at time-point 1, representing 69.4% of the total variation. This reduced to seven principal components at time-point 2, which represented 62.8% of the total variation. Models of the non-progressor group with stage 1 OA (Figure 3b) were more stable, with six principal components meeting the threshold at both time-points 1 and 2, accounting for 43.4% and 45.0% of the total variation, respectively.

For the progressor group (Figure 3c), at time-point 1 (stage 1 OA), six principal components met the 5% threshold, accounting for 42.0% of the total variation. However, as these samples progressed to stage 2 OA at time-point 2, this number dropped to five principal components, and their combined contribution to the total variation decreased to 35.4%.

### 3.1 Longitudinal analysis

Non-parametric combination tests revealed no differences in the models of the non-progressor groups between time-points 1 and 2 for those with stage 0 (p = 0.999) and stage 1 OA (p = 0.986). In contrast, differences were found in the models for those in which OA progressed from stage 1 to stage 2 OA (p < 0.001).

**FIGURE 4:**
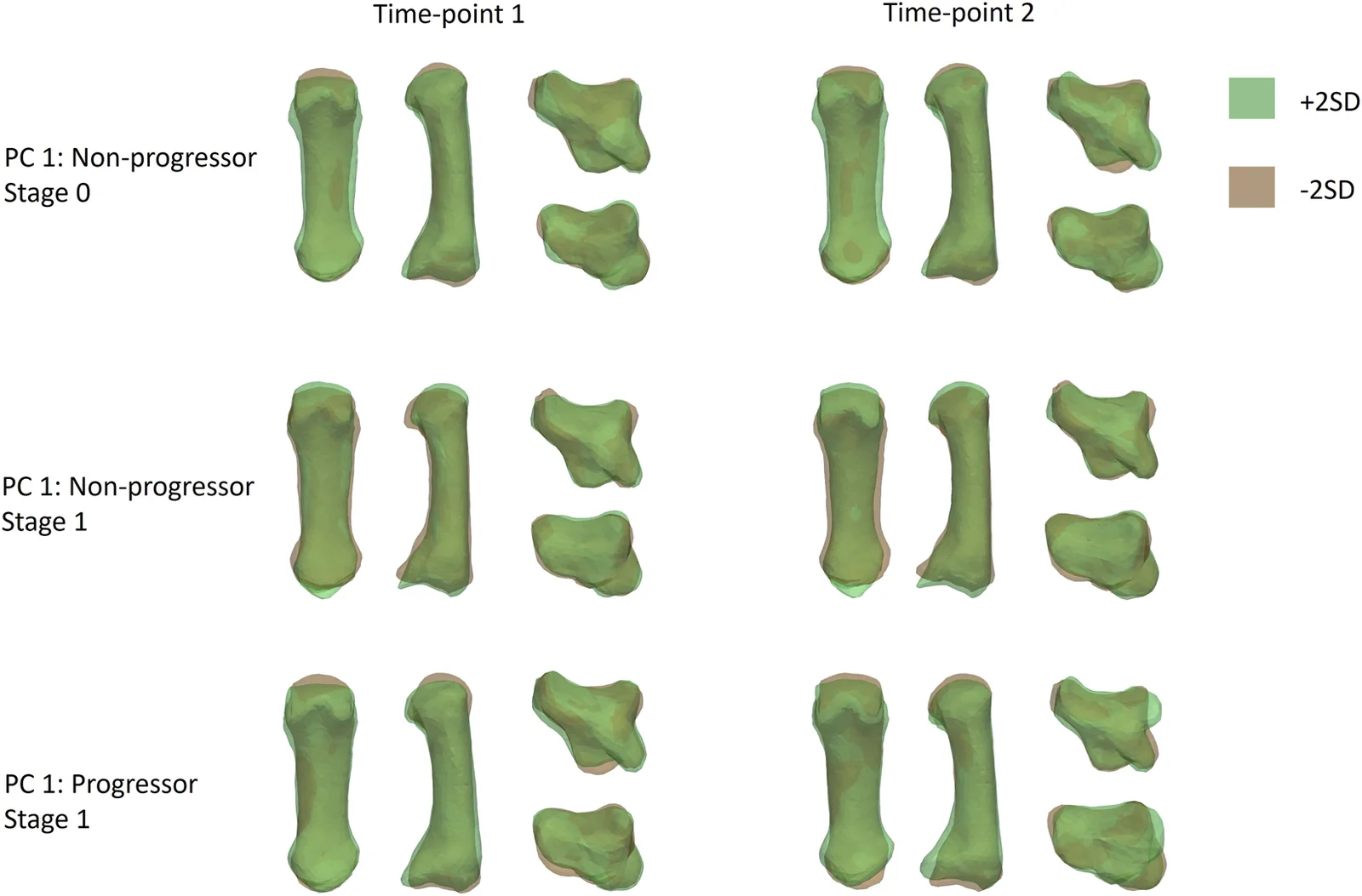
The primary mode of morphological variation was determined by the first principal component (PC1), across the groups at both time-points. Each individual panel displays an overlay of the shape model between the +2 standard deviations (SD) (green) and −2 SD (brown). The morphological variation patterns of the non-progressor groups appear to remain consistent over the entire follow-up period. Conversely, the progressor group exhibits noticeable shifts in dominant morphological variance.

**FIGURE 5:**
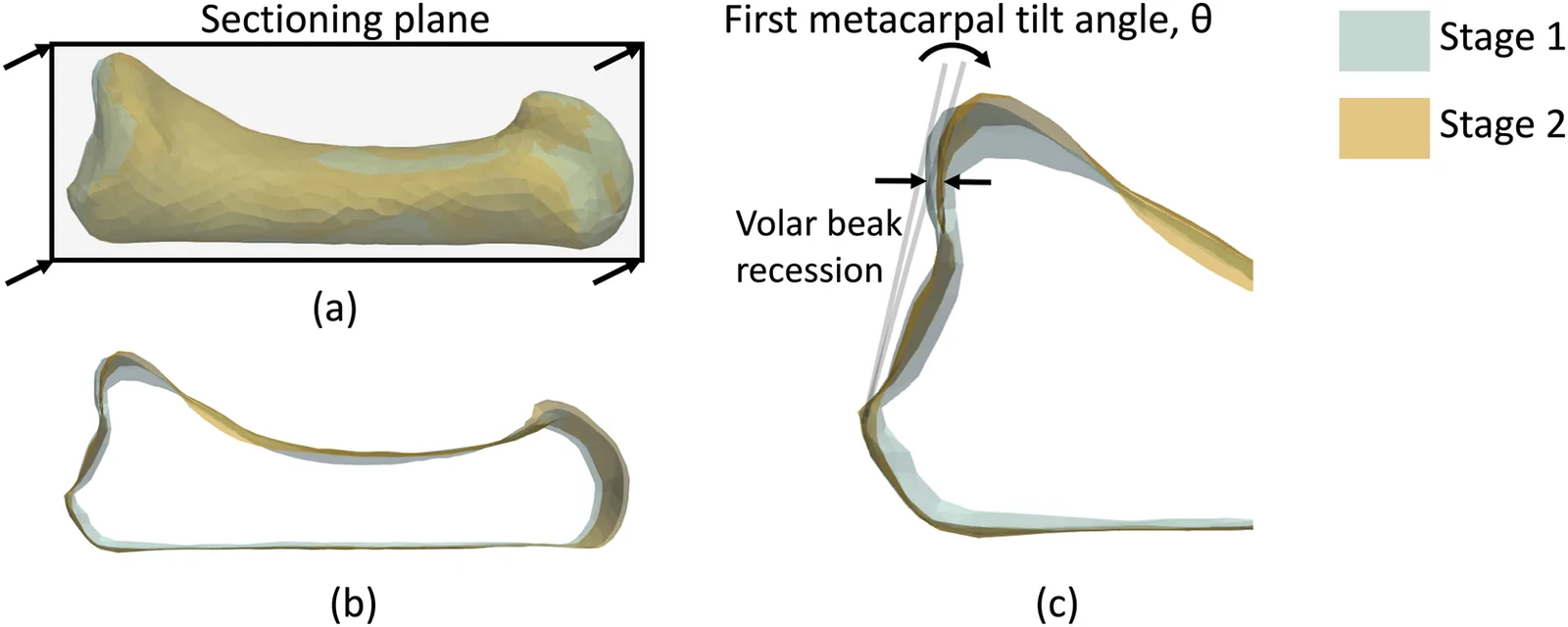
The longitudinal morphological changes within the first metacarpal of the progressor group, which highlights morphological deformations over the follow-up interval. **(a)** The sectioning plane used to cut both the baseline and follow-up mean shape models for morphological analysis. The stage 1 baseline is shown in teal, while the stage 2 follow-up is shown in orange. **(b)** The exposed internal geometry of the first metacarpal that allows the visualisation of the volar beak. **(c)** View of the volar beak recession affecting the first metacarpal tilt angle (θ).

**FIGURE 6:**
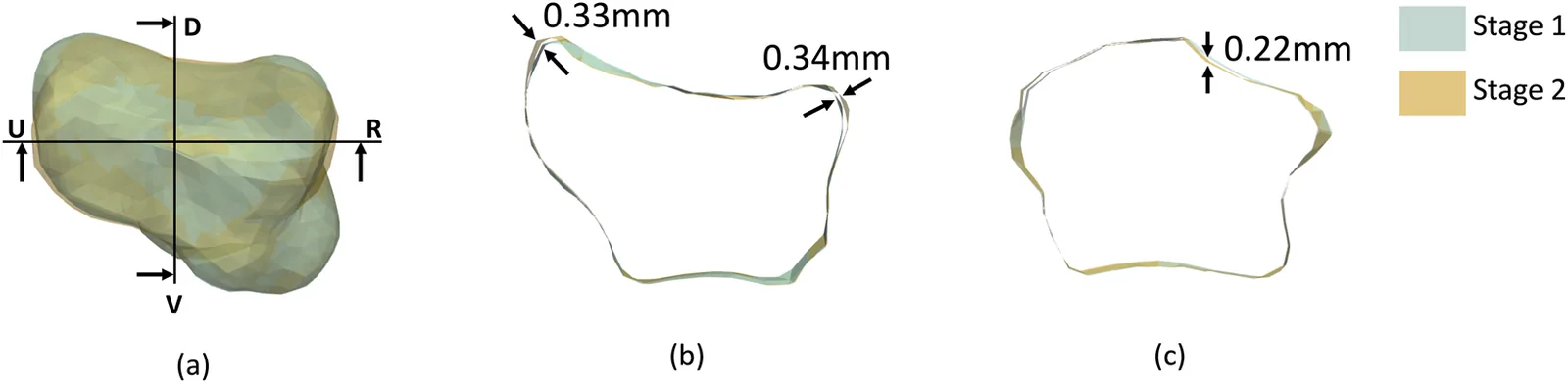
The longitudinal morphological changes within the trapezium of the progressor group, which highlights morphological deformations over the follow-up interval. **(a)** The dorsal-volar (D–V) and ulna-radial (U–R) sectioning planes used to cut both the baseline and follow-up mean shape models for morphological analysis. The stage 1 baseline is shown in teal, while the stage 2 follow-up is shown in orange. **(b)** U-R view highlighting the progressive protrusions along the first carpometacarpal facet. These expansions measure 0.34 mm and 0.33 mm at the radial and ulnar borders. **(c)** D-R view indicating a corresponding 0.22 mm geometric depression, highlighting the collapse during osteoarthritis progression.

Because principal component analysis orthogonally ranks statistical variance, the first principal component captures the highest degree of morphological variation observed within the entire group. This implies that by analysing the first principal component, a direct assessment of dominant morphological changes involving the longitudinal tracking of these specific morphological characteristics can be seen. For the non-progressor group, the shape models obtained indicated that the pattern of morphological variation remained stable between time-points 1 and 2 (Figure 4), reflecting the outcomes obtained from the statistical analysis. As OA progressed, the progressor group showed morphological changes when compared to its baseline for both the first metacarpal and trapezium (Figure 4). Localised morphological changes were observed at the proximal end of the first metacarpal, specifically at the volar beak. Visual inspection indicated these changes were recession of the first metacarpal volar beak, which leads to the distal progression of the first metacarpal tilt angle (Figure 5). Morphological changes on the trapezium between time-points 1 and 2 were observed at the radial and ulnar border of the first carpometacarpal facet (Figures 6a,b). A depression was observed at the volar region of the first CMC joint articulating surface on the trapezium near the volar carpometacarpal facet (Figures 6a,c).

### 3.2 Progressor vs. non-progressor analysis

At time point 1 for the non-progressor group, no differences were obtained for those with stage 0 and stage 1 OA (p = 0.993). However, differences were found between those whose OA progressed and those whose did not (p < 0.001) at time-point 1.

**FIGURE 7:**
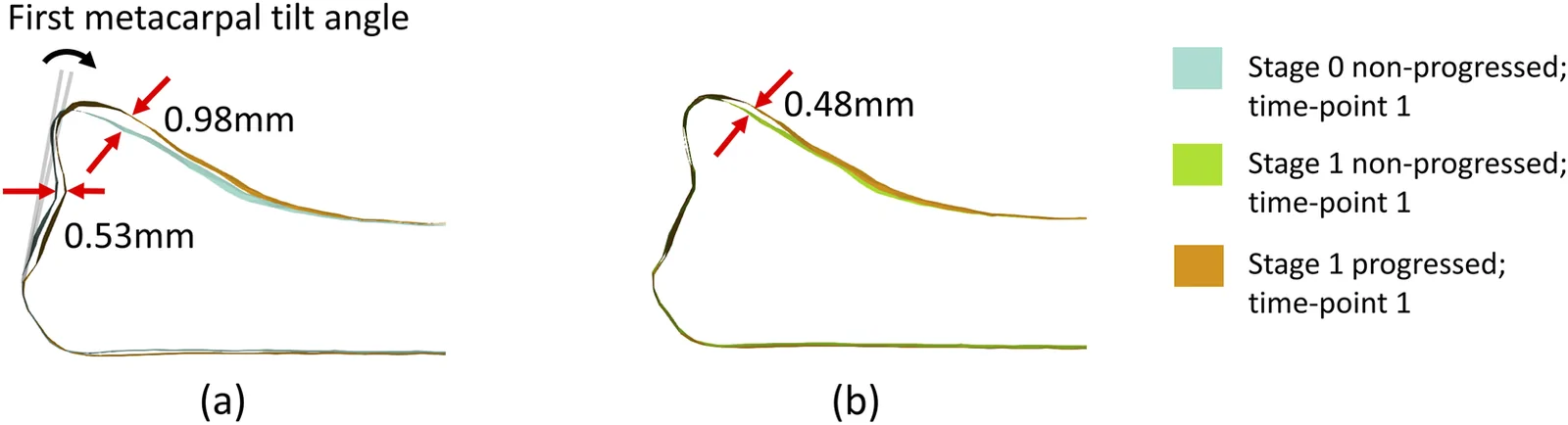
A cross-sectional inter-group comparison of the first metacarpal. **(a)** The progressor stage 1 (orange) shape model is directly overlaid against the non-progressor stage 0 group (teal), demonstrating a 0.98 mm protrusion of the volar beak and 0.53 mm recession of the first carpometacarpal articulating surface. **(b)** The progressor stage 1 group (orange) is directly overlaid against the non-progressor stage 1 group (green), demonstrating a 0.48 mm protrusion of the volar beak.

The mean shape models demonstrated that the first metacarpal tilt angle for the progressor group was observed to be more distal than that of the stage 0 non-progressor group (Figure 7a). In addition, when compared to both stage 0 and stage 1 non-progressor groups, a more pronounced protrusion of the volar beak on the mean shape model of the progressor group was observed (Figure 7).

**FIGURE 8:**
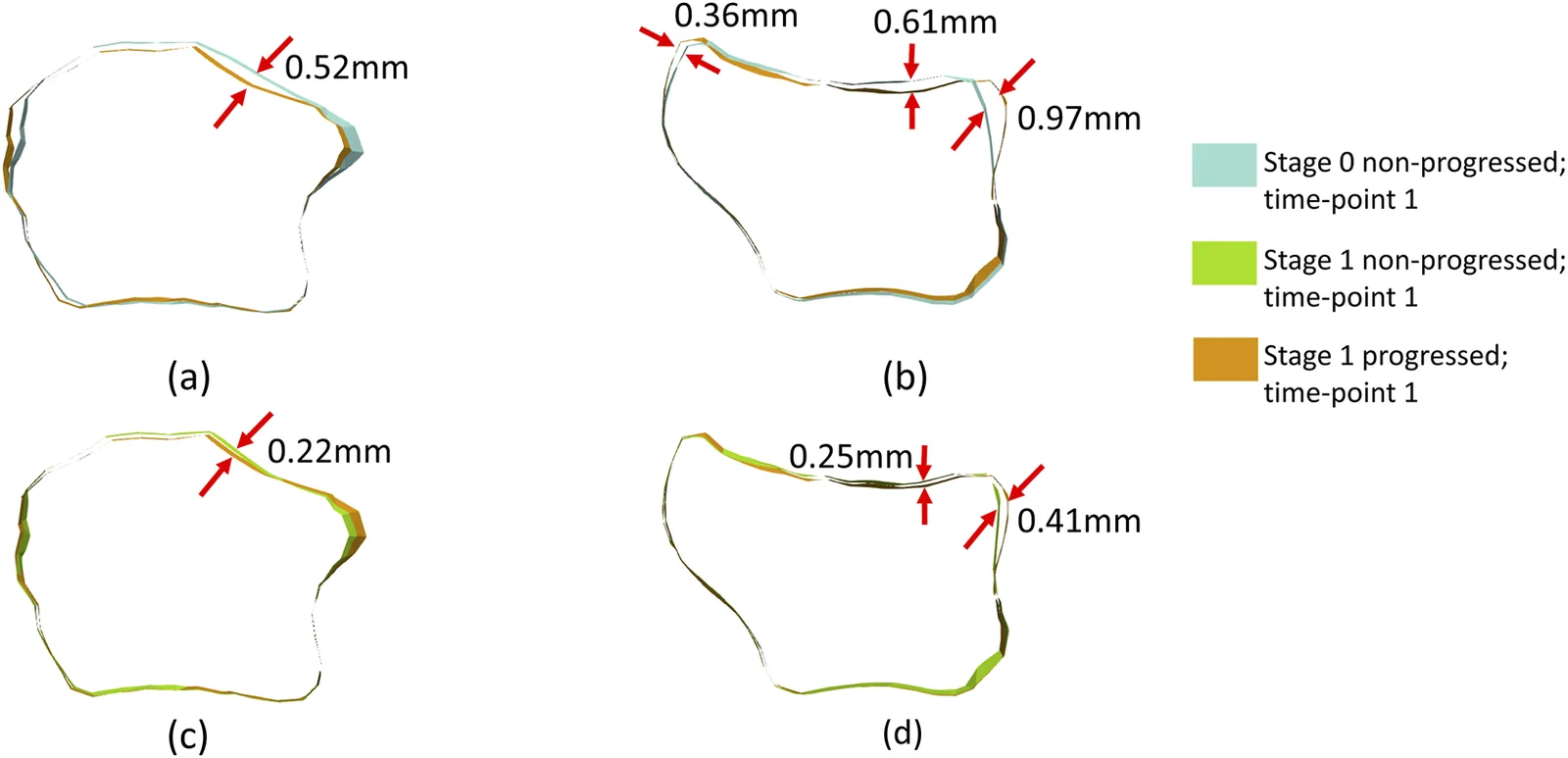
A cross-sectional inter-group comparison of the trapezium to evaluate baseline data (time-point 1) and identify early morphological markers predictive of disease trajectory. The progressor stage 1 (orange) shape model is directly overlaid against **(a,b)** the non-progressor stage 0 (teal), and **(c,d)** non-progressor stage 1 (green) groups. Dorsal-volar (D–V) sectional views highlight the early morphological changes within the progressor group, indicated by a morphologic depression along the volar articulating surface across **(a,c)**. The ulnar-radial (U–R) view in **(b,d)** indicates protrusions at the radial and/or ulnar borders.

The mean shape model of the progressor group trapezium indicates a depression in the volar region of the articulating surface near the CMC facet (Figures 8a,c). The same morphological trait was observed on the trapezium mean model in the radial region of the articulating surface near the radial border of the CMC facet (Figures 8b,d). However, this morphological trait in the progressor group was more prominent when compared to that of the stage 0 non-progressor group. The progressor group trapezium mean model also showed the protrusion at the radial border of the carpometacarpal facet (Figures 8b,d). This morphological trait was also observed at the ulnar border when compared to the stage 0 non-progressor group but, not visible when compared to the stage 1 group that did not progress.

## 4 Discussion

This study was conducted to discern whether the morphology of the first metacarpal and trapezium, could provide information regarding the potential for progression of the first CMC joint OA. To investigate this, this study utilised a longitudinal dataset with variable follow-up durations of either 1.5- or 6-year post baseline. The 6-year scan was prioritised for analysis; however, for participants without a 6-year post baseline scan, the 1.5-year scan was utilised.

This study employed SSM to quantify longitudinal changes in the first CMC joint morphology within a cohort of early-stage OA patients. Statistically, this study has proven that the morphology of the first metacarpal and trapezium differ between participants with first CMC joint OA that progressed and participants with first CMC joint OA that did not progress. This aligns with previous research indicating that trapezium dimensions (height, width, and volar recession) and the first metacarpal features (volar beak recession and angle) vary between these groups (Morton et al., 2022). Morphological analysis was done qualitatively on the obtained shape models to identify anatomical parameters that differentiate the first CMC joint OA patients that progressed with non-progressed patients.

The analysis of the principal component variation over the follow-up period revealed distinct morphological behaviours across the cohorts. The non-progressor group exhibited stability in its morphological variation, with patterns remaining almost unchanged over the follow-up period. This suggests an absence of the active pathological remodelling characteristic of advancing OA, indicating a ‘stable disease’ state. In contrast, the progressor group displayed noticeable instability, with the relative contributions of its principal components shifting significantly over time. While the non-progressor stage 0 OA cohort was also relatively stable, it did demonstrate minor shifts in its morphological variation. These slight alterations are not indicative of pathology but are likely attributable to the normal biological drift of aging, including ongoing bone remodelling and biomechanical adaptation to loading. Taken together, these findings strongly suggest that the temporal instability of morphological variation patterns, rather than a static shape profile alone, may serve as a key quantitative biomarker to distinguish progressive OA from both stable disease and the natural morphological changes of healthy aging.

The progressor group demonstrated that OA progression is associated with an increased distal progression of the first metacarpal tilt angle, a change corresponding to increased flatness of the first metacarpal volar beak. Reduction in the concavity of the volar beak may cause the incongruence of the joint, which may result in instability of the first CMC joint. This may explain the dorsal subluxation phenomenon that is commonly observed with the first CMC joint OA patients. Previous studies highlighted that first CMC joints with OA have a smaller first metacarpal tilt angle than healthy first CMC joints (Miura et al., 2004; Kurosawa et al., 2013). A smaller tilt angle of the first metacarpal could increase the shear force that translates the first metacarpal dorsally (Miura et al., 2004). Dorsal subluxation during functional tasks like thumb flexion, opening a jar, and pinch have been described to potentially be used as predictors of disease progression for early-stage OA (Morton et al., 2023). The dorsal subluxation allows compression force at the first CMC joint to be concentrated at the first metacarpal volar beak (Kurosawa et al., 2013). This condition is supported by previously reported findings mentioned that cartilage degeneration frequently starts in the volar region of the first metacarpal (Koff et al., 2003).

When compared to the stage 0 OA non-progressor group, the progressor group was observed to have a prominent depression of the articulating surface near the volar region of the carpometacarpal facet. The protrusion observed in the progressor group at the first metacarpal volar beak might be an indicator of the early formation of osteophytes, which could be the result of abnormal loading experienced by the joint. This morphological trait observed in the progressor group may not cause the progression of the OA but may be the result of cartilage degeneration. As this volar beak protrusion was not observed in the stage 0 OA non-progressor group, this morphological trait may be an early indicator of the progression of first CMC joint OA.

The principal components highlighted that morphological variations of the trapezium were observed at the volar ridge of the first metacarpal facet on the trapezium. The morphology of the convex surface of the first metacarpal facet on the trapezium can be important as this anatomical feature may be used to compensate for the morphological variation of the first metacarpal volar beak. Marzke et al. suggested that a small first metacarpal tilt angle can cause the first metacarpal volar beak to abut with the convex surface of the first metacarpal facet on the trapezium (Marzke et al., 2012). Thus, the arcuate volar ridge of the trapezium observed in this study may result in the avoidance of bony impingement of the first metacarpal volar beak.

Demographic variables, such as age and gender, represent population level correlates of hand and first CMC joint OA prevalence (van der Oest et al., 2021). However, the present study did not adjust for these covariates, and current evidence does not establish how these demographic variables alter SSM-derived morphological progression. Although sex differences in scaphotrapeziotrapezoid joint morphometrics have been demonstrated (Trentadue et al., 2025) and higher OA prevalence among women is well documented (van der Oest et al., 2021; Joko et al., 2025), direct evidence linking these factors to morphological-based progression markers is presently absent. Therefore, any interpretation of demographic effects on morphological progression must be viewed as speculative.

There are a few limitations in this study that is worth mentioning. First, the duration between time-points 1 and 2 was not the same for all samples. However, this combination of samples with different durations between two time-points was done to increase the samples in each of the datasets, making the shape model more representative of the population under study. Another limitation was that the number of samples in each dataset was not the same. However, the authors believe that this did not affect the shape models, as the number of samples used in this study was consistent with other studies of a similar nature (Schneider et al., 2015; Schneider et al., 2018). Furthermore, the root-mean-squared error obtained from the leave-one-out analysis indicates that the statistical shape model possesses acceptable generalisability. Finally, this study is dependent on the static 3D imaging of the thumb in its neutral alignment. Consequently, this study lack of *in vivo* kinematic or kinetic data to confirm the biomechanical implications of the morphological findings. The mechanical consequences are inferred based on static spatial features and existing literature rather than directly measured dynamic forces. Future investigations incorporating dynamic imaging techniques or subject-specific musculoskeletal modelling are necessary to validate the relationship between the longitudinal morphological variations and *in vivo* joint mechanics.

## 5 Conclusion

Morphology of both the first metacarpal and trapezium obtained using statistical shape modelling was able to differentiate between the first CMC joints of OA patients that progressed to a higher stage from those that did not progress. Understanding how the morphology of the first metacarpal and trapezium varies and changes over time can be beneficial in the diagnosis and decision-making regarding treatment and rehabilitation pathways for first CMC joint OA.

## Data Availability

The raw data supporting the conclusions of this article will be made available by the authors, without undue reservation.
